# Adenovirus-Associated Disseminated Intravascular Coagulation

**DOI:** 10.7759/cureus.14194

**Published:** 2021-03-30

**Authors:** Syed Ather Hussain, Aneeqa Zafar, Hafsa Faisal, Olga Vasylyeva, Farhan Imran

**Affiliations:** 1 Internal Medicine, Rochester Regional Health, Rochester, USA; 2 Internal Medicine, El Camino Hospital, Mountain View, USA; 3 Infectious Diseases, Rochester Regional Health, Rochester, USA; 4 Hematology Oncology, Rochester Regional Health, Rochester, USA

**Keywords:** adenovirus, coagulopathy, disseminated intravascular coagulation, ocps

## Abstract

A 21-year-old previously healthy Caucasian female presented to the emergency department (ED) in the pre-COVID-19 era for evaluation of thrombocytopenia after a flu-like illness. The patient reported fever, cough, headache and myalgias for one week. She was on oral contraceptive pills (OCPs) for five years but discontinued one week ago. She was found to be in disseminated intravascular coagulation (DIC) and her hospital course was complicated by intraparenchymal hemorrhage, deep vein thrombus (DVT) in the right arm veins, bilateral pulmonary embolus (PE) and multiple splenic infarcts. An extensive workup was negative but nasopharyngeal swab came back positive for adenovirus by polymerase chain reaction (PCR).

## Introduction

Adenoviruses are amongst the most common causes of respiratory viral illnesses and typically resolve without complications [1]. Adenoviruses can rarely cause fatal meningoencephalitis, myocarditis and disseminated intravascular coagulation (DIC) [1,2]. We report a case of a young immunocompetent female who suffered from adenoviral pneumonia with progressive clinical deterioration towards DIC followed by a complete recovery.

## Case presentation

A 21-year-old Caucasian female was referred to the emergency department (ED) by her primary care physician (PCP) for evaluation of thrombocytopenia after a flu-like illness. The patient reported fever, cough, headache and myalgias of one-week duration. She had no personal or family history of thrombocytopenia. She denied smoking, vaping, alcohol or recreational drug use. She was on oral contraceptive pills (OCPs) for five years but discontinued one week ago by herself as she did not feel well. She was not on any other medications. Her vitals were within normal limits. Physical exam was only remarkable for right upper extremity (RUE) warmth and swelling. Platelet count was 24x103/µL, white count 11.9x103/µL and hemoglobin 10.5 g/dL. The pregnancy test was negative. Overnight, her hemoglobin dropped to 5.0 g/dL. Coagulation profile showed prothrombin time 14.9 sec, activated partial thromboplastin time 25.8 sec, fibrin split products > 20 µg/mL, D-dimer > 7,650 FEUng/mL and fibrinogen 68 mg/dL. Erythrocyte sedimentation rate (ESR) was 10 mm/hr. Peripheral smear showed schistocytes. Her haptoglobin was 318 mg/dL and lactate dehydrogenase 600 U/L.

**Figure 1:**
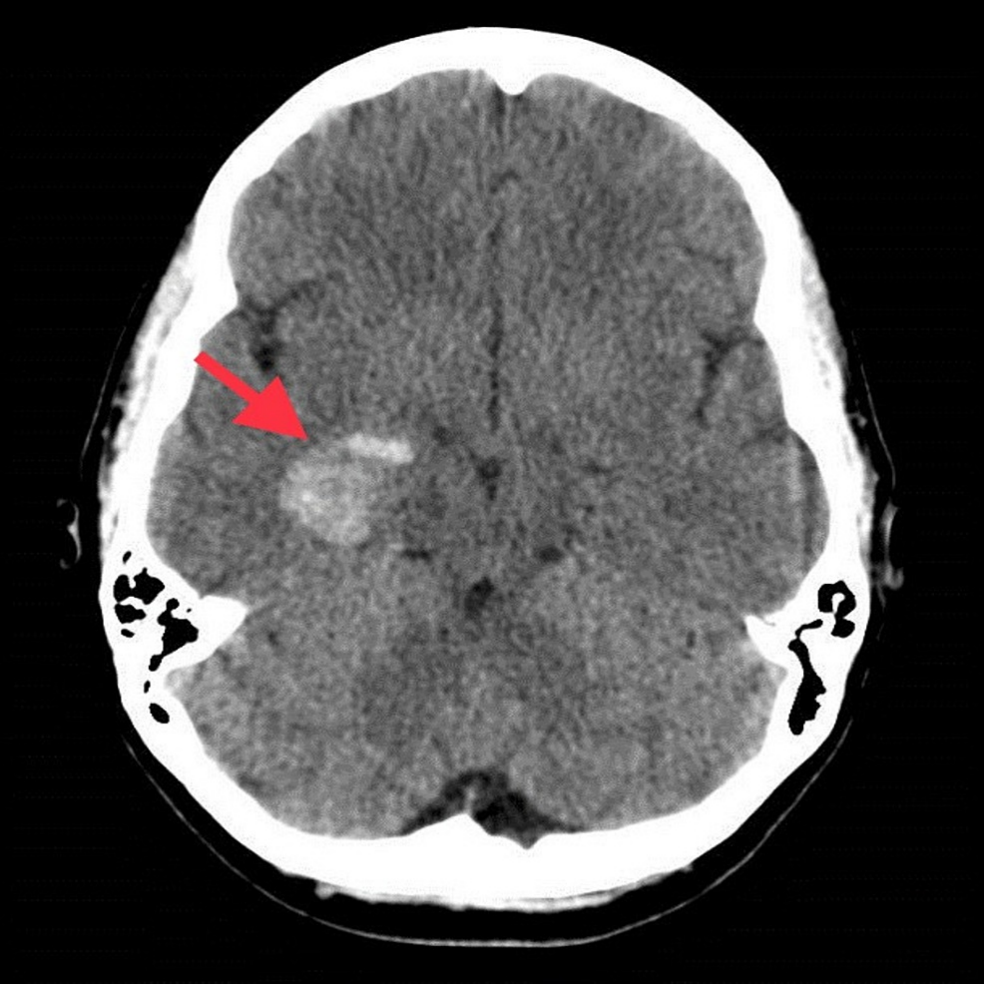
Intraparenchymal bleed in the medial right temporal lobe measuring 2.2 cm in diameter without midline shift

**Figure 2:**
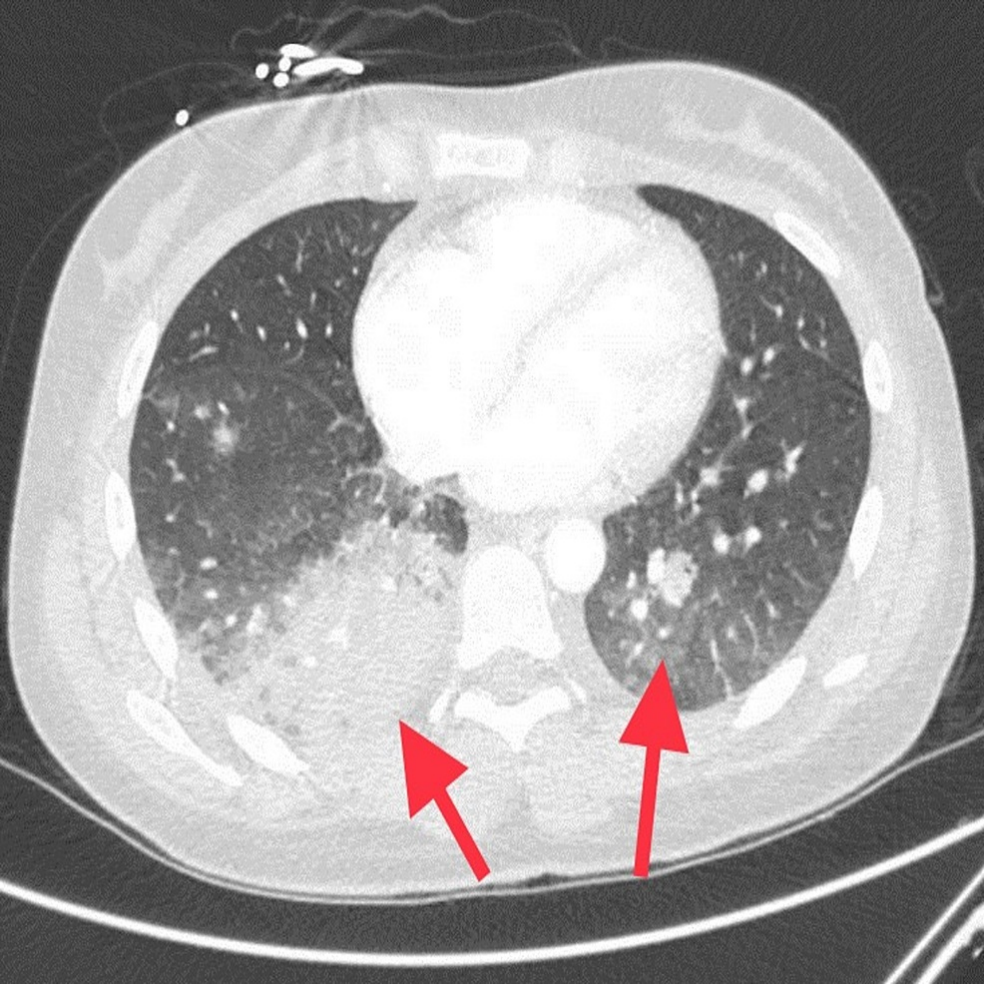
Large area of consolidation in the right lower lobe with patchy airspace opacification in the left lower lobe

**Figure 3:**
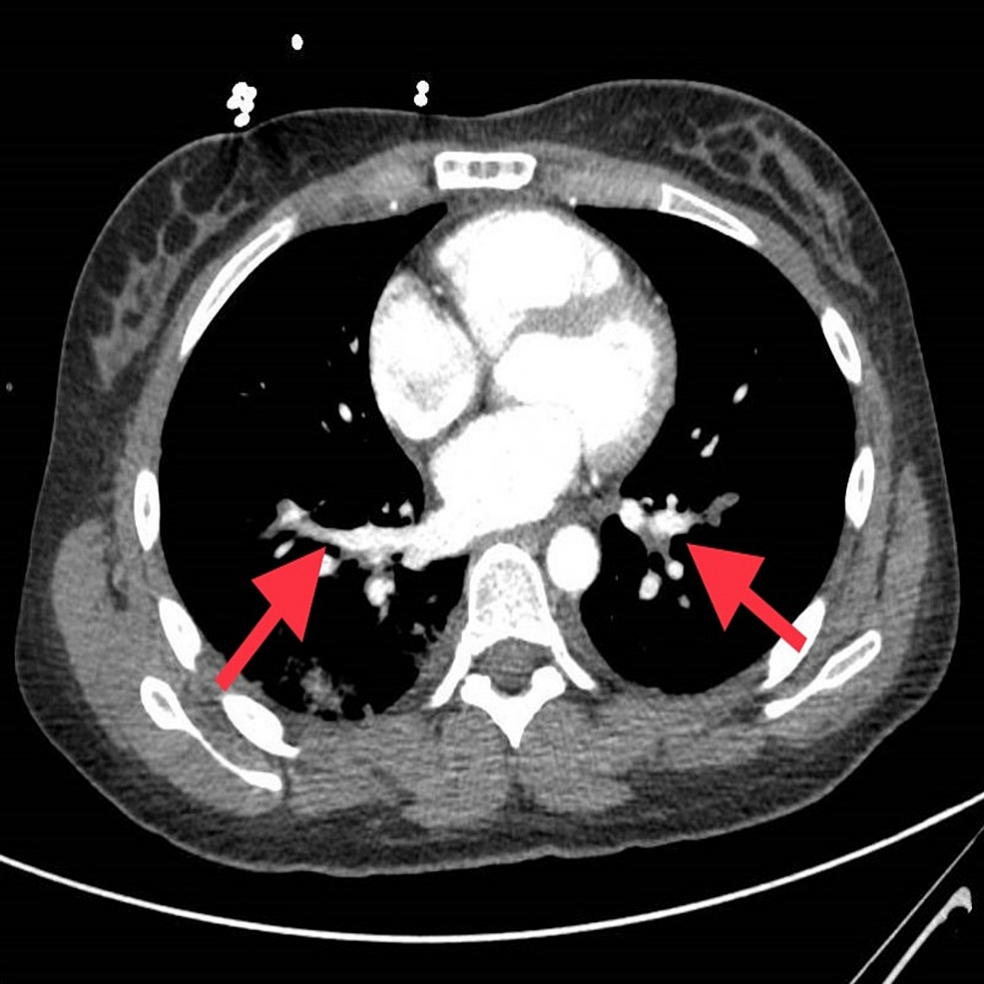
Small amount of pulmonary artery embolus in the lobar branch of the left lower lobe and in the right lower lobe

**Figure 4:**
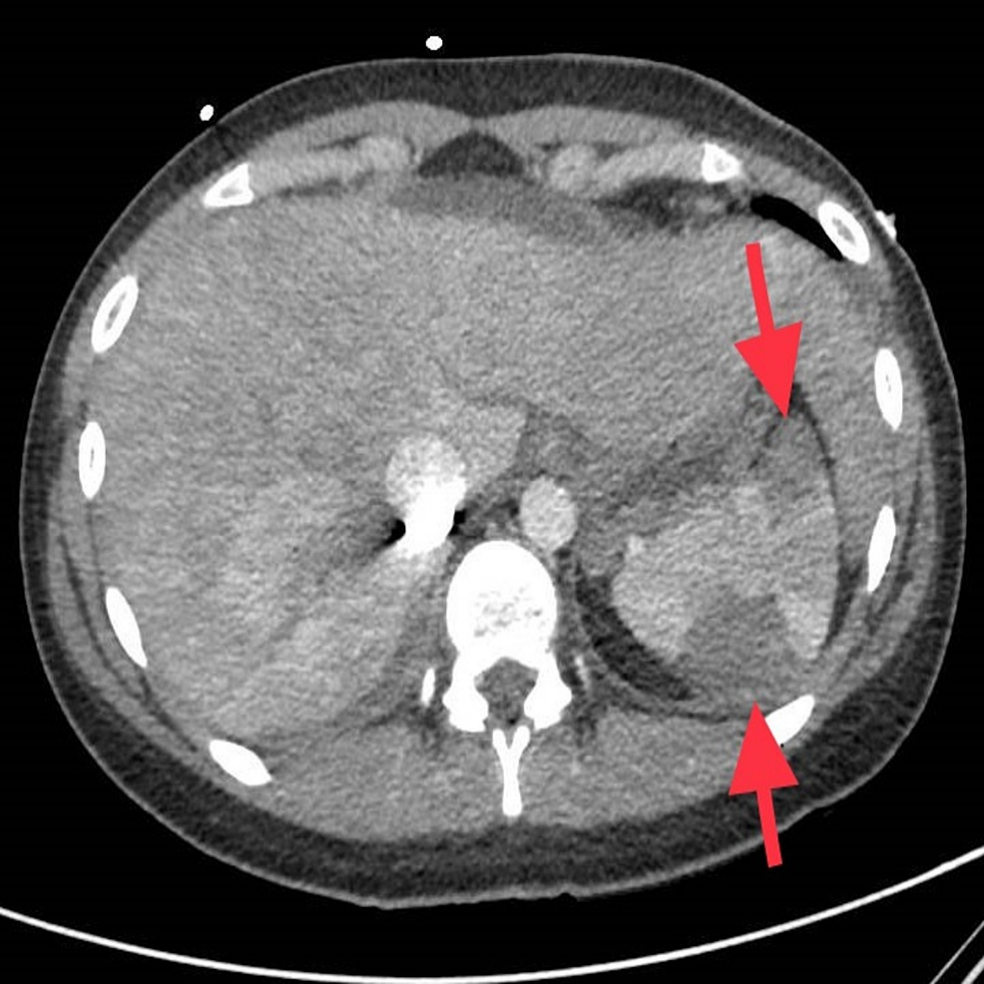
Multiple peripheral wedge-shaped infarcts in the spleen

RUE ultrasound revealed deep vein thrombus (DVT) in the right axillary, brachial and basilic veins. She underwent computed tomography (CT) head which showed intraparenchymal hemorrhage (Figure 1). Hematology recommended intravenous methylprednisolone, cryoprecipitate, and fresh frozen plasmas (FFPs), after which her serum fibrinogen improved to 160 mg/dL. Neurosurgery recommended no intervention. CT chest showed right lower lobe and patchy left lower lobe consolidation (Figure 2). She also had small pulmonary embolus (PE) in the right and left lower lobes (Figure 3). CT abdomen and pelvis showed multiple splenic infarcts (Figure 4) and she was started on empiric antibiotics and heparin.

Antinuclear, anticardiolipin, beta-2-glycoprotein, heparin-platelet factor 4 and ADAMTS13 inhibitor antibodies, as well as paroxysmal nocturnal hemoglobinuria (PNH), JAK2 and prothrombin gene mutation testing were all negative. ADAMTS13 levels were within normal limits.

Procalcitonin was 0.12 ng/mL. Respiratory, blood, urine and stool cultures were negative. Methicillin-resistant Staphylococcus aureus (MRSA) nasal swab was negative. Echocardiogram revealed no valvular abnormalities. Group A Streptococcus throat culture, monospot test, cytomegalovirus serum viral load, HIV, hepatitis B and C, Legionella urine antigen, Anaplasma, Ehrlichia chaffeensis, parvovirus B19 and Lyme disease serology all returned negative. Nasopharyngeal viral polymerase chain reaction (PCR) came back positive for adenovirus and was negative for coronavirus (SARS-CoV-2 strain was not included in the assay at the time), metapneumovirus, rhinovirus/enterovirus, influenza A and B, parainfluenza, respiratory syncytial virus, Bordetella pertussis, Chlamydia pneumoniae and Mycoplasma pneumoniae.

DIC was thought to be triggered by adenovirus infection. She was discharged home on rivaroxaban 10 mg daily, which she continues to take to date. She reports doing well at one year follow up. She had normal protein C, S, and antithrombin levels. Lupus anticoagulant could not be tested as a patient remains on anticoagulation.

## Discussion

Adenoviruses have the ability to infect the endothelium based on a review of 84 autopsies [2]. Endothelial cell infection leads to increased tissue factor expression on its surface, which is mediated by inflammatory cytokines like interleukin (IL)-1, IL-6 and tumor necrosis factor (TNF). This in turn causes a state of systemic inflammatory response, further cell injury and coagulopathy [3]. It has been hypothesized that immunocompromised state can lead to sufficient infectious adenoviral particles to build up in the bloodstream allowing endothelial infection; however, our patient had no evidence of immunosuppression [2].

Interestingly, our patient was taking OCPs until a week prior to the presentation. OCPs are well known to cause thromboembolic disease but not DIC. It is possible that the OCPs created a background hypercoagulable state, and a superimposed adenovirus infection triggered a cascade of thrombosis and hemorrhage.

The year 2020 has highlighted COVID-19 as the leading cause of viral coagulopathy [4]. Our patient presented three months before the first case of COVID-19 was confirmed in the United States, and she had no history of traveling outside the country. In the present era dominated by COVID-related coagulopathy, it is important to remember the lesser known.

## Conclusions

Adenoviruses can be a rare and unusual cause of DIC in immunocompetent individuals. We performed a thorough work up and were unable to identify any other etiology for DIC in our patient. We aim to raise awareness that in the era dominated by COVID-19 and hemorrhagic viral infections, the seemingly benign adenovirus should not be overlooked as a potential cause of DIC. More research is required to understand possible interactions between OCP use and concomitant viral infection.
